# Supplementary material for: Chiroptical spectroscopy of a freely diffusing single nanoparticle
Source: Nat Commun. 2020 Sep 9;11:4513. doi: 10.1038/s41467-020-18166-5 (PMC7481242; doi:10.1038/s41467-020-18166-5)
Supplement: Supplementary file 1 — Supplementary Information [file 41467_2020_18166_MOESM1_ESM.pdf]

Supplementary Information

Chiroptical Spectroscopy of a Freely Diffusing Single Nanoparticle

Sachs and Günther et. al

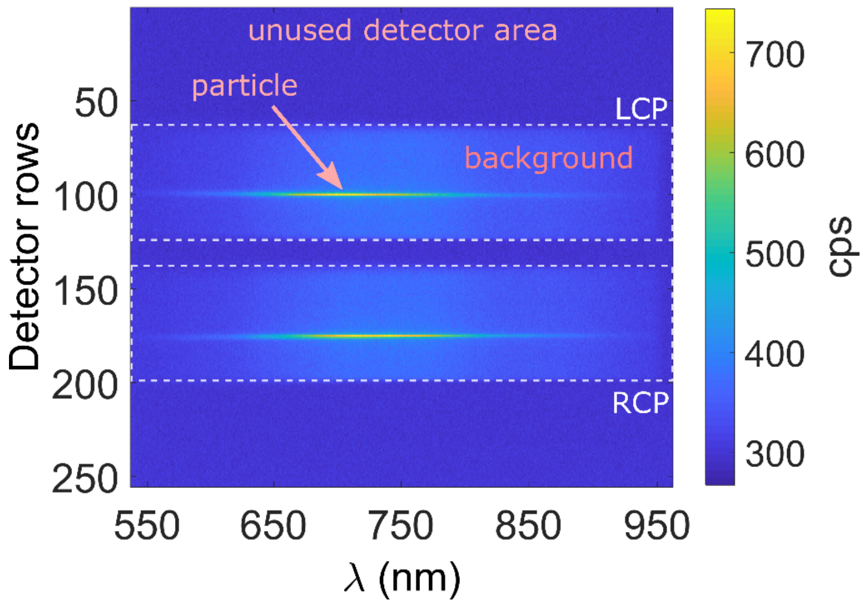

Supplementary Figure 1: Snapshot of the CCD showing the raw signal. Particles can be distinguished from the low intensity background due to stronger localized intensities. Spatially separated particles appear on different rows of the CCD. This permits the identification of a scatterer of interest. The particle concentration is kept dilute enough to ensure that the chance of several particles entering the field of view is very low.

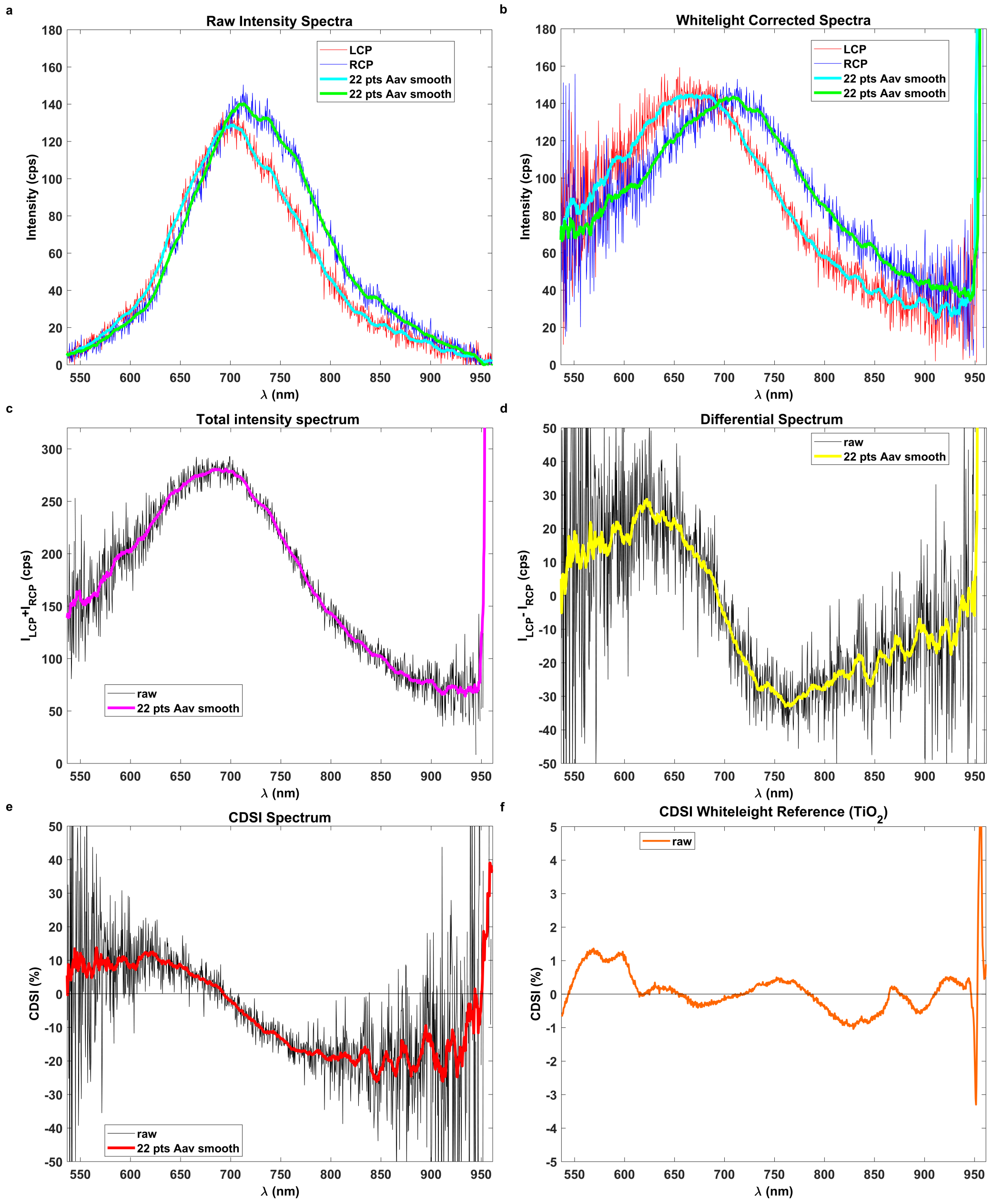

Supplementary Figure 2: Scattering spectra from a LH nanohelix for one individual frame with an exposure time of 1 s. (a) Raw signal, (b) Divided by TiO_2 reference, (c) Total scattering intensity $I_{LCP} + I_{RCP}$, (d) Unnormalized differential scattering $I_{LCP} - I_{RCP}$, (e) CDSI as defined in the main text and (f) uncorrected CDSI of the TiO_2 reference, which defines the instrument's response.

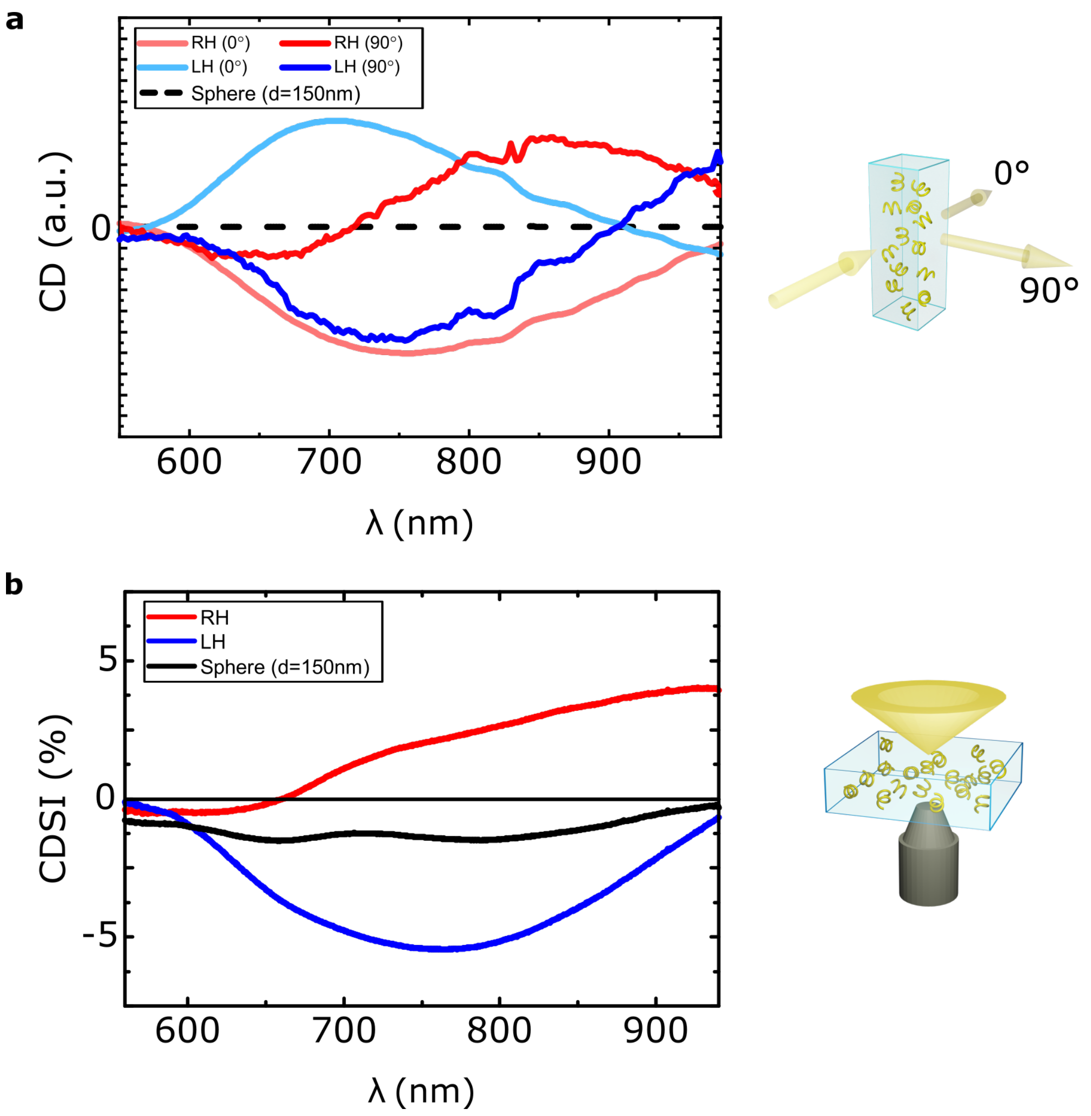

Supplementary Figure 3: Ensemble-averaged chiroptical spectroscopy $\langle \mathrm{CDSI} \rangle_N$ in a classical cuvette experiment and in the dark-field spectrometer (both in H_2O). (a) Usual CD measurement in transmission ($\theta = 0°$) records attenuation of the light and show zero CD for achiral spheres, whereas for nanohelices peaks of opposite sign are observed for opposite handedness (scaled on y-axis). By mounting the detector at $\theta \approx 90°$, scattering intensities are recorded and the sign of the nanohelix spectrum is reversed and shifted to longer wavelengths by ~ 50 nm. (b) Ensemble averaged CDSI measured with the dark-field spectrometer (corresponding to SI-Fig.4a-c) are acquired at a different scattering angle but come to a similar result.

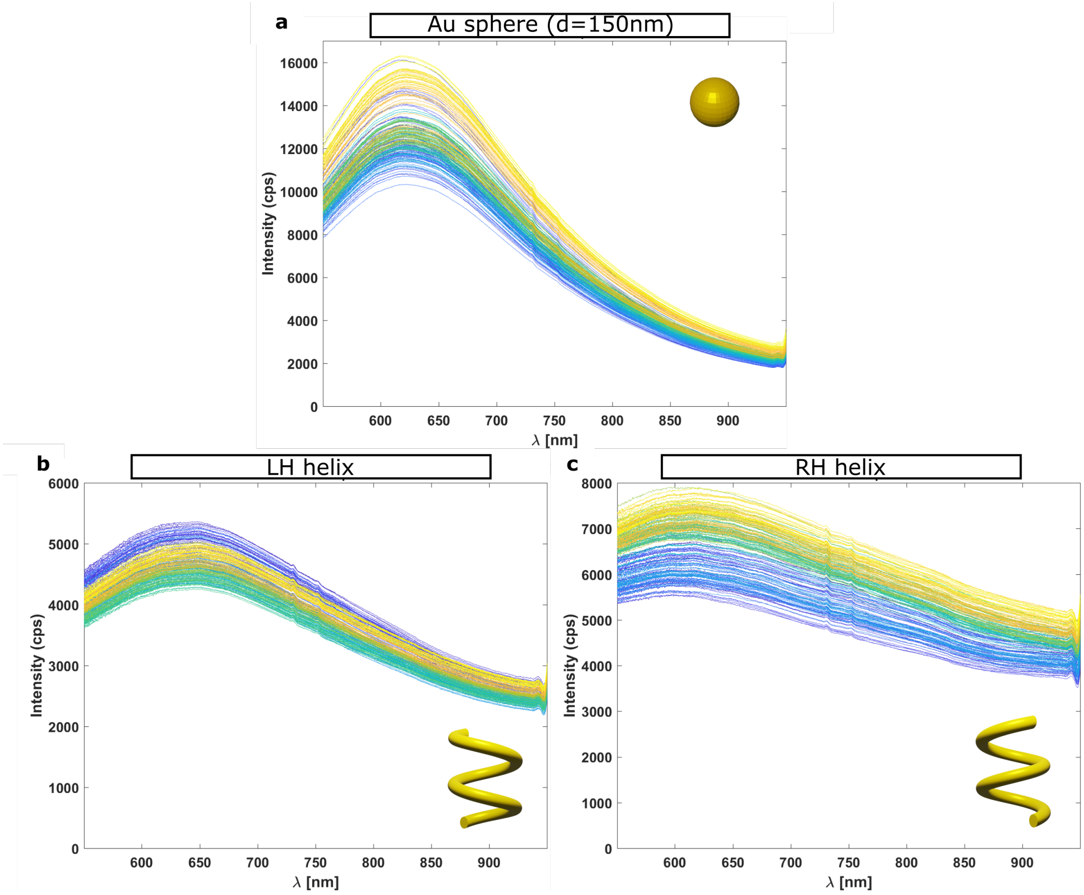

Supplementary Figure 4: Ensemble measurements of commercial samples as well as nanostructures prepared by PVD recorded with our dark-field scattering spectroscopy setup. The total scattering intensity averaged over 20 minutes (3 s exposure time) is obtained for (a) Au nanospheres with diameter $d = 150$ nm, (b) LH Au nanohelices and (c) RH Au nanohelices.

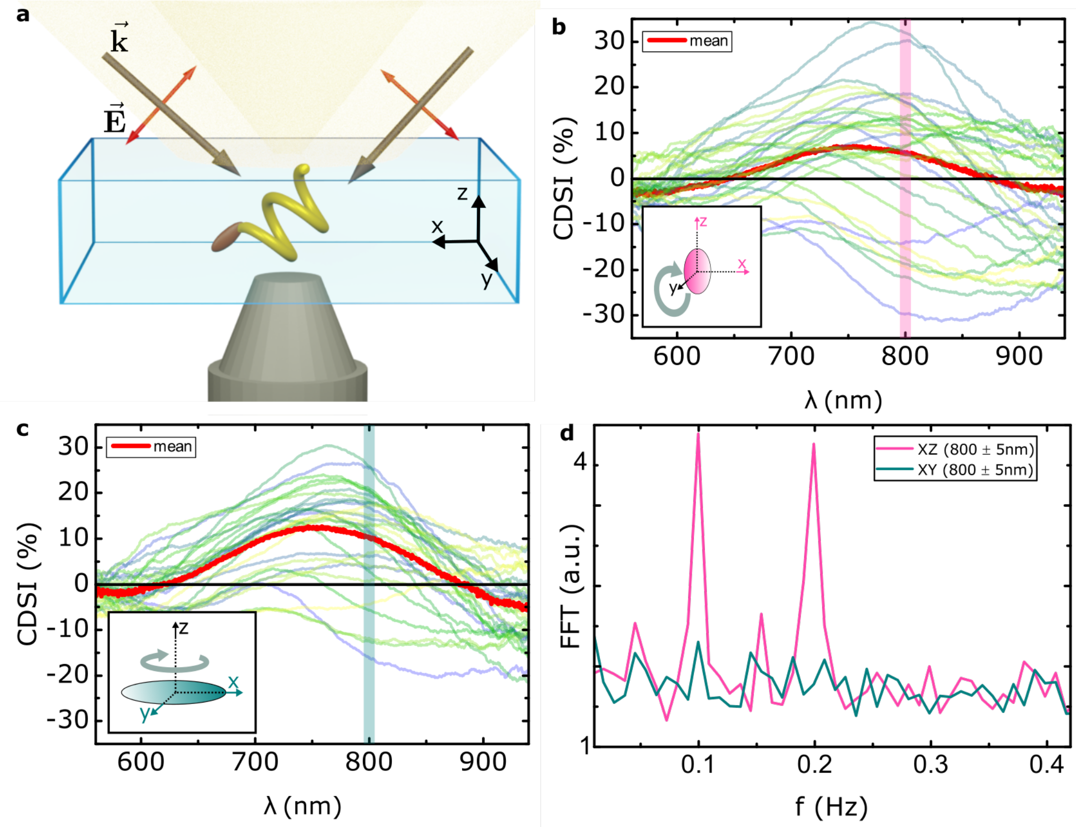

Supplementary Figure 5: Externally induced motion of the magneto-plasmonic helix in a magnetic field that is rotated in one plane only. a) Incident light with electric field vector $\vec{\mathbf{E}}$ illuminates the helix under a fixed scattering angle. b) CDSI spectra acquired at different instants in time of the Au nanohelix (RH) rotating in the xz-plane and c) for a rotation in the xy plane show distinct fluctuation patterns. d) By calculating the Fourier transform of the fluctuating intensities in (b) and (c) at $\lambda = 800 \pm 5$ nm either two or no peaks are identified depending on the symmetry between the incident light and the scatterer and hence they indicate an in-sync rotation with the external magnetic field.

Supplementary Note 1: Evaluation Workflow of Time-Series Single-Particle Spectroscopy

The CCD detector in our dark-field scattering spectroscopy setup has 255 rows, each with a height of 26 µm. The column dimension of the detector is used for spectral separation. Since we used a 40x objective, each row on the CCD corresponds to a rectangular area with a width of 650 nm in the sample plane. The size of the nanostructures is on the order of $d \approx 150$ nm. It is thus reasonable to assume that only a few adjacent rows on the detector capture the spectrum of a single particle. The other detector rows will collect light stemming from other scatterers in the field of view as well as impurities in the vicinity. Such effects contribute to a pale intensity distribution, which is used for a background correction (see SI-Fig.1). Prior to a spectral acquisition, an individual particle was selected and centered by moving the microscope stage. Single particles could be distinguished from agglomerates due to the vastly differing scattering intensities. Multiple particles in the field of view were readily identified by the corresponding peaks registered at different positions on the CCD.

Because the nanoparticles can freely diffuse, the maximum measurement time $t_{\max}$ for different particles and viscosities can be estimated with the Brownian mean square displacement. The dimensions of the aperture set a spatial constraint $\Delta x = 5$ μm in the object plane: $\langle \Delta x^2 \rangle = 2Dt_{\max}$. Hence, a 150 nm diameter nanosphere can be observed for approximately 25 minutes (1:20 glycerol), whereas in water ($\eta \approx 1$ mPas) it will leave the observation volume after approximately 4 seconds. In general, the nanoparticles can change their position (row on the CCD) between subsequent acquisitions. The first step in the evaluation of a frame in the time series was therefore to locate the position of the particle on the CCD array. For this we used an automatic custom peak finding routine implemented in MATLAB. The number of rows contributing to the particle spectrum was held constant during the evaluation of the time-series measurement. The raw data deduced from a single frame randomly taken from the measurement of a LH nanohelix is shown in SI-Fig.2a. Since the particles move, we set the exposure time for one frame

to 1 s (cycle time 1.1048 s). This value corresponds to a good trade-off between a fast enough acquisition and a suitably long time for a good signal-to-noise ratio. Nevertheless, the measured data is still noisy as shown in SI-Fig.2a, but the shape of the curve can be seen. We used the raw data for all of our calculations. However, in order to plot several curves in one figure, we smoothed the data, with an adjacent-averaging algorithm with an interval of 22 or 55 neighbouring wavelength data points, corresponding to intervals of 9.13 nm and 22.8 nm. This was used in several plots of the main text and in SI-Fig.2a-e. In SI-Fig.2 the smoothed curves are overlaid onto plots of the raw/unsmoothed data.

The recorded raw spectra in SI-Fig.2a are still convolved with the spectrum of the illumination source, the quantum efficiency of the detector, the spectral response of the optical components (waveplates, mirrors, etc.). To access the pure scattering spectrum originating only from the particle, the data needs to be deconvolved. We use a 1.5 μm TiO_2 bead as a spectrally flat scatterer for our whitelight reference measurement. A normalization is performed by dividing the LCP and RCP spectra acquired for the probe by the response from the reference measurement (see SI-Fig.2b). Those signals are then used to further calculate the total scattering intensity (SI-Fig.2c) or the difference (SI-Fig.2d) of the two circular intensities. The latter signal is sometimes used to show a chiral dissymmetry in other studies. However, because the illumination source intensity fluctuates over time this difference needs to be normalized by the total intensity to obtain an absolute value. This normalization is essential to permit comparison between different frames or between different samples.

Supplementary Note 2: Angle-Dependence of Chiroptical Spectroscopy

Circular dichroism (CD) spectroscopy is a classical chiroptical spectroscopy. It measures the difference in attenuation between left- and right-circularly polarized light transmitted through a cuvette containing the analyte. The absorption is described by the Lambert-Beer law. The differential intensities are acquired at a scattering angle of $\theta = 0°$. However, in general, both extinction and scattering are always present and contribute to the circular intensity difference[1]:

$$\mathrm{CD} = \frac{I_\mathrm{L} - I_\mathrm{R}}{I_\mathrm{L} + I_\mathrm{R}} = \frac{-2.303\,(\varepsilon_\mathrm{L} - \varepsilon_\mathrm{R})\,c\ell}{2} + \frac{\sigma_\mathrm{L}(0) - \sigma_\mathrm{R}(0)}{2r^2 + \sigma_\mathrm{L}(0) + \sigma_\mathrm{R}(0)}. \tag{1}$$

Here c is the concentration of the sample, l the distance the light propagates through the probed medium, r the distance between cuvette and detector, and $\sigma(\theta)$ the angle-dependent scattering cross-section of the medium. ε is the extinction of the sample, generally stemming from absorption and scattering $\Delta\varepsilon = \varepsilon_\mathrm{L} - \varepsilon_\mathrm{R} = (A_\mathrm{L} - A_\mathrm{R}) + (S_\mathrm{L} - S_\mathrm{R})$. For molecules the experiments occur in the Rayleigh limit with negligible scattering ($S_{L,R} \approx 0$) and in practice only absorption is significant for the attenuation of the light propagating through the cuvette with the sample. Thus, extinction reduces to $\Delta\varepsilon = A_\mathrm{L} - A_\mathrm{R}$ and usually only the first term in (1) is considered in CD spectroscopy:

$$\mathrm{CD} = \frac{I_\mathrm{L} - I_\mathrm{R}}{I_\mathrm{L} + I_\mathrm{R}} \simeq \frac{-2.303\,(\varepsilon_\mathrm{L} - \varepsilon_\mathrm{R})\,c\ell}{2} \propto A_\mathrm{L} - A_\mathrm{R} \tag{2}$$

On the contrary, for nanoparticles scattering plays an important role and cannot be neglected. Thus, the circular differential signal is highly dependent on the scattering geometry, i.e., the distance r and the scattering angle θ. Additionally, if the differential intensities for cp-light are acquired at any other angle except zero, only scattered light is detected. Hence, the circular differential scattering intensity is described by[1]:

$$\mathrm{CDSI} = \frac{I_\mathrm{L} - I_\mathrm{R}}{I_\mathrm{L} + I_\mathrm{R}} = \frac{-2.303\,(\varepsilon_\mathrm{L} - \varepsilon_\mathrm{R})\,c\ell}{2} \left[\frac{(1+\cos\theta)^2}{2\,(1+\cos^2\theta)}\right] + \frac{\sigma_\mathrm{L}(\theta) - \sigma_\mathrm{R}(\theta)}{\sigma_\mathrm{L}(\theta) + \sigma_\mathrm{R}(\theta)}. \tag{3}$$

Classical CD experiments in a commercial spectrometer utilize a cuvette and a transmission geometry such that Eq.(1) applies. However, if the detector is mounted at an angle

other than $\theta = 0°$, then scattering intensities are also recorded (see Eq.3). The same is true in our dark-field setup where the condenser's and objective's NA (1.2-1.4 and 0.9) fix the scattering angle to lie between ($\theta = 17° - 110°$) and hence, here scattering dominates[2,3]. Consequently, measuring the CDSI of an ensemble in our setup is related to the classical ensemble CD (at $\theta \neq 0°$) but not equivalent to it, because the scattering angle differs. Notice, that for achiral samples the differential intensity is zero in any geometry.

To test this, colloidal solutions of nanoparticles (in H_2O) were measured in a commercial spectrometer (JASCO J-810) in transmission ($\theta = 0°$) as well as in a geometry where the detector was mounted at $\theta \approx 90°$ with respect to the incident light direction. The results are shown in SI-Fig.3a with a scaled y-axis for the latter measurement. In fact, the detected signal was too low to reliably determine the ensemble averaged scattering CDSI at $\theta \approx 90°$. However, qualitatively a change in sign and shift of the CDSI peaks by ~ 50 nm was observed when comparing to the classical CD measured in transmission at $0°$. The sign change is anticipated when one considers that e.g. a left-handed nanoparticle absorbs and scatters more LCP than RCP light. This leads to intensities $I_{\mathrm{R}}^{Abs} > I_{\mathrm{L}}^{Abs}$ when absorbance is measured ($\theta = 0°$), whereas for scattering ($\theta \neq 0°$) the recorded intensities yield $I_{\mathrm{L}}^{Scat} > I_{\mathrm{R}}^{Scat}$. It follows that in the aforementioned geometries the sign of $I_L - I_R$ is reversed depending whether absorption or scattering intensities are recorded. Most noteworthy, achiral spheres posses zero circular intensity differences at any detection angle, because of their isotropic shape. Additionally, the data obtained by the commercial instrument is in agreement with the ensemble-averaged CDSI measurements in our dark-field scattering setup, see SI-Fig.3b (detailed description in Supplementary Note 3).

Consequently, the question whether a sample is chiral can be deduced from both the classical CD experiment in a cuvette or a CDSI measurement obtained from a dark-field spectrometer. Either by measuring an ensemble-average of many particles (SI-Fig.3) or by averaging the CDSI of a single particle over a long enough observation time, during which its orientations are isotropically sampled (Fig.1a of the main text). In fact, the ensemble-average yields broadened spectral measurements compared to the single-

particle experiment, because any nanoparticle ensemble shows polydispersity in shape and size. However, if the shape variations are small, then the ergodicity of the system is still valid: $\langle \mathrm{CDSI} \rangle_t \approx \langle \mathrm{CDSI} \rangle_N$. This has been demonstrated and is shown by the agreement between Fig.2d and e of the main text.

Supplementary Note 3: Acquisition of Ensemble-Averages in the Dark-Field Spectrometer

To compare the single particle time-average $\langle\mathrm{CDSI}\rangle_t$ with ensemble averages $\langle\mathrm{CDSI}\rangle_N$ the CDSI of particle ensembles in solution (H_2O) are measured in the dark-field spectrometer and in a cuvette utilizing a commercial CD spectrometer (JASCO J-810). The former involves averaging over an area of $\sim 40\ \mu\mathrm{m}\times 20\ \mu\mathrm{m}$ in the sample plane and using sufficiently dense solutions. The translational diffusion constant for Brownian motion of the nanoparticles in H_2O is $\sim 3\ \frac{\mu m^2}{s}$. Each frame was recorded with an exposure time of 3 s, which ensures an average over tens of particles per frame. By acquiring a sequence of 400 frames with a total measurement duration of 20 minutes, it is reasonable to assume that good ensemble averaging can also be obtained in the dark-field spectroscopy setup. The same samples from the main text were measured: commercial Au nanospheres (diameter $d = 150$ nm) and the right- and left-handed nanohelices grown by PVD as described in the main text. The total scattering intensity for these samples, as observed with our setup, is shown in SI-Fig.4a-c. As expected from theory[4] we see a fairly broad resonance for the spherical gold nanoparticles around $\lambda = 650$ nm. For the left- and right-handed nanohelices we also observe resonance peaks. The LH helices display a more distinct peak while the RH helices show a broadened peak that could be due to a less homogeneous structure or other imperfections during the PVD growth process.

The mean $\langle\mathrm{CDSI}\rangle_N$ ($t = 400$ frames) for the sphere and the two helix enantiomers are shown in SI-Fig.3b. The achiral spheres have zero CDSI up to a constant offset while the chiral helices display peaks of opposite sign, as expected. In accordance with the total intensity spectra, the CDSI of the RH helix sample appears broadened and slightly shifted towards longer wavelengths (beyond the spectral range accessible by our instrument). As anticipated the ensemble-averages acquired with our dark-field setup qualitatively agree with the results from the commercial CD spectrometer (SI-Fig.3a) because they are related (but not equal).

Supplementary Note 4: Validation of Magnetically Induced Orientation

In order to conduct the experiment with magneto-plasmonic helices shown in Fig.4 of the main text, we first validated that the particles can be controlled by the external magnetic field. Therefore, we rotated the field vector along a circular trajectory, such that the long axis of the nanohelix rotated with a fixed frequency ($f_{ext} = 0.1$ Hz) in one plane. We moved the helix in the xz plane and in the xy plane according to the geometry visualized in SI-Fig.5a. The first 30 frames of the time-series measurements (total duration $t = 100$ s) are shown in SI-Fig.5b and c, respectively. One can immediately recognize a difference in individual CDSI spectra as well as their average value. Even more apparent is the fluctuation of the CDSI at $\lambda = 800 \pm 5$ nm (not shown here). If we analyse the fluctuation with a fast fourier transformation (FFT), as can be seen SI-Fig.5d, we find two characteristic frequencies for the rotation in the xz plane, and no characteristic frequency for the rotation in the xy plane. This can be explained by the geometry of the annular dark-field condenser, which illuminates the nanoparticle with a hollow light cone (SI-Fig.5a) and thus in the former case the helix is constantly changing its alignment with respect to the electric field vector of the incident light field. Remember that the finite helix has C_1 symmetry. The CDSI thus changes systematically when its axis is parallel or orthogonal to the xy plane, and once more depending which end of the helix points up. Therefore we anticipate a peak at twice the driving frequency ($2f_{ext}$) and one at f_{ext}. These correspond to the two peaks in the FFT of SI-Fig.4d at $f = 0.1$ Hz and $f = 0.2$ Hz. In contrast, while rotating the helix's long axis parallel to the xy plane its alignment with respect to the incident light (electric field vector) is always the same, irrespective of its orientation in xy. Changes observed in the CDSI are thus due to statistical fluctuations only (e.g. tilt out of xy plane) and average to zero in the FFT. Our results demonstrate that the nanoparticle's orientation is indeed controlled by the magnetic field and that it is co-rotating in sync with the external field.

Supplementary Note 5: Mueller-Stokes Analysis of the Setup

Light propagating through an optical element can be modeled mathematically by utilizing the Mueller calculus. It describes the polarization state of the electric field by a 4-component vector and optical elements by 4x4 Mueller matrices M. The propagation through the setup is given by matrix multiplication $\mathrm{S}_{out} = \mathrm{M} \cdot \mathrm{S}_{in}$, or explicitly:

$$\mathrm{S_{out}} = \begin{bmatrix} s'_0 \\ s'_1 \\ s'_2 \\ s'_3 \end{bmatrix} = \begin{bmatrix} m_{00} & m_{01} & m_{02} & m_{03} \\ m_{10} & m_{11} & m_{12} & m_{13} \\ m_{20} & m_{21} & m_{22} & m_{23} \\ m_{30} & m_{31} & m_{32} & m_{33} \end{bmatrix} \begin{bmatrix} s_0 \\ s_1 \\ s_2 \\ s_3 \end{bmatrix} . \quad (4)$$

The propagation through a series of $\mathbf{n}$ elements is a multiplication of $\mathbf{n}$ matrices

$$\mathrm{M}_{\mathbf{Total}} = \mathrm{M}_{\mathbf{n}} \cdot ... \cdot \mathrm{M}_{\mathbf{3}} \cdot \mathrm{M}_{\mathbf{2}} \cdot \mathrm{M}_{\mathbf{1}}. \quad (5)$$

In our case the light scattered by a single nanoparticle exits the microscope and is then incident on a quarterwaveplate (QWP) at +45°, a Wollaston prism at 0° and a second QWP at -45°. The prism spatially separates light corresponding to the two linear polarization states. Afterwards they are spectrally resolved by a grating and are captured on two non-overlapping locations on the CCD detector (see Fig.1c of main text). It is therefore reasonable to model our optical train as two individual channels and the Wollaston prism as a linear polarizer (LP) at 0° for channel one and as a LP at 90° for the second channel. Hence, the resulting Mueller matrices for the two channels are:

$$\begin{aligned} \mathrm{M_L} &= \mathrm{M}_{\mathbf{QWP}}(-\mathbf{45}^\circ) \cdot \mathrm{M}_{\mathbf{LP}}(\mathbf{0}^\circ) \cdot \mathrm{M}_{\mathbf{QWP}}(+\mathbf{45}^\circ), \\ \mathrm{M_R} &= \mathrm{M}_{\mathbf{QWP}}(-\mathbf{45}^\circ) \cdot \mathrm{M}_{\mathbf{LP}}(\mathbf{90}^\circ) \cdot \mathrm{M}_{\mathbf{QWP}}(+\mathbf{45}^\circ). \end{aligned} \quad (6)$$

The exact form of the matrices can be found elsewhere[5]. Since our setup detects intensities

(photon flux), we focus on the first component of the Stokes vector only:

$$I_{\mathrm{L,R}} = [1, 0, 0, 0] \cdot \mathbf{M}_{\mathrm{L,R}} \cdot \begin{bmatrix} s_0 \\ s_1 \\ s_2 \\ s_3 \end{bmatrix}, \tag{7}$$

By inserting (6) into (7) we derive $I_{\mathrm{L}} = s_0 + s_3$ and $I_{\mathrm{R}} = s_0 - s_3$ for the intensities in the two channels representing scattered LCP and RCP light, respectively. The CDSI can then be calcuated:

$$\mathrm{CDSI} = \frac{I_{\mathrm{L}} - I_{\mathrm{R}}}{I_{\mathrm{L}} + I_{\mathrm{R}}} = \frac{s_3}{s_0}. \tag{8}$$

Hence, our instrument measures the s_3 component of the Stokes vector, which is equivalent to the *degree of circular polarization*[5].

1. Bustamante, C. et al., Circular differential scattering can be an important part of the circular dichroism of macromolecules. *Proceedings of the National Academy of Sciences* **80**, 3568–3572 (1983).

2. Wang, L.-Y. et al., Circular differential scattering of single chiral self-assembled gold nanorod dimers. *ACS Photonics* **2**, 1602–1610 (2015).

3. Karst, J. et al., Single plasmonic oligomer chiral spectroscopy. *Advanced Optical Materials* **6**, 1800087 (2018).

4. Bohren, C.F. and Huffman, D.R., Absorption and Scattering of Light by Small Particles, Appendix A: Homogeneous Sphere, 477–482. *John Wiley & Sons, Ltd*, (2007).

5. Bass, M. et al., Handbook of optics, Volume II: Design, fabrication and testing, sources and detectors, radiometry and photometry. *McGraw-Hill, Inc.* (2009)
